# MALDI-TOF Mass Spectrometry and Specific Biomarkers: Potential New Key for Swift Identification of Antimicrobial Resistance in Foodborne Pathogens

**DOI:** 10.3390/microorganisms7120593

**Published:** 2019-11-21

**Authors:** Maureen Feucherolles, Henry-Michel Cauchie, Christian Penny

**Affiliations:** 1 Luxembourg Institute of Science and Technology (LIST), Environmental Research and Innovation (ERIN) Department, 41 rue du Brill, 4422 Belvaux, Luxembourg; henry-michel.cauchie@list.lu; 2 Faculté des Sciences, de la Technologie et de la Communication (FSTC), Doctoral School in Science and Engineering (DSSE), University of Luxembourg, 2 avenue de l’Université, 4365 Esch-sur-Alzette, Luxembourg

**Keywords:** MALDI-TOF MS, biomarkers, foodborne pathogens, antimicrobial resistance, diagnostics

## Abstract

Matrix-assisted laser desorption/ionization time of flight mass spectrometry (MALDI-TOF MS) is today the reference method for direct identification of microorganisms in diagnostic laboratories, as it is notably time- and cost-efficient. In the context of increasing cases of enteric diseases with emerging multi-drug resistance patterns, there is an urgent need to adopt an efficient workflow to characterize antimicrobial resistance (AMR). Current approaches, such as antibiograms, are time-consuming and directly impact the “patient-physician” workflow. Through this mini-review, we summarize how the detection of specific patterns by MALDI-TOF MS, as well as bioinformatics, become more and more essential in research, and how these approaches will help diagnostics in the future. Along the same lines, the idea to export more precise biomarker identification steps by MALDI-TOF(/TOF) MS data towards AMR identification pipelines is discussed. The study also critically points out that there is currently still a lack of research data and knowledge on different foodborne pathogens as well as several antibiotics families such as macrolides and quinolones, and many questions are still remaining. Finally, the innovative combination of whole-genome sequencing and MALDI-TOF MS could be soon the future for diagnosis of antimicrobial resistance in foodborne pathogens.

## 1. The Burden of Antimicrobial Resistances Worldwide: The Case of Foodborne Pathogens

**Figure 1:**
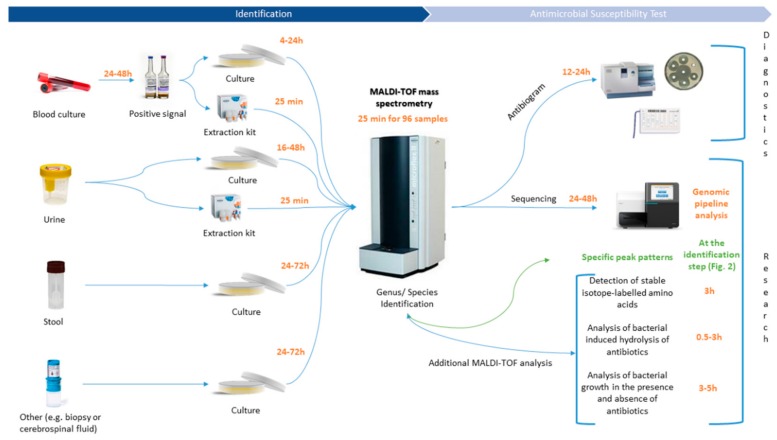
MALDI-TOF MS related analysis workflow in clinical routine diagnostic and research laboratories.

For decades, antibiotics have been increasingly used in human and veterinary medicine, to treat bacterial infections such as gastrointestinal, respiratory or urinary tract infections and septicemia [1]. Drugs of veterinary importance are not only used for therapeutic purposes, but also as a preventive measure (metaphylaxis and prophylaxis) and growth promoter [2]. Hence, selected resistances within pathogens appear along the food chain with most often humans as the final hosts. Likewise, antibiotics overuse and inappropriate prescribing are other main reasons for bacterial genetic adaptation and exchange facing selective pressure [3]. These mechanisms are naturally present in microbial communities among various ecosystems, such as aquatic systems [4]. Nowadays, antimicrobial resistance (AMR) is considered a major threat to global public health by its influence on human health and the related economic issues. According to a report from the Organization for Economic Cooperation and Development (OECD), infections by resistant microorganisms will cause 2.4 million deaths in Europe, North America and Australia in the next 30 years and cost up to $3.5 billion per year [5]. As well, a World Health Organization (WHO) report highlighted a total of 349 million registered foodborne illnesses and 187,285 deaths caused by bacteria worldwide in 2010 [6]. Among these pathogens, *Acinetobacter* spp., *Bacillus* spp., *Campylobacter* spp., *Citrobacter* spp., *Clostridium* spp., *Enterobacter* spp., *Escherichia* spp., *Klebsiella* spp., *Listeria* spp., *Salmonella* spp., *Shigella* spp., *Staphylococcus* spp., *Vibrio* spp. and *Yersinia* spp are the main causes of such diseases [7]. Specifically, foodborne pathogens are in an ever-increasing focus due to the emergence of multi-drug resistance patterns worldwide. Studying and understanding interfaces between human health, animal health and the environment seems to be a requirement to understand the circulation of AMR among the food chain [8]. The “One Health” approach combines various disciplines to ensure optimal health for humans, animals, wildlife, plants and the environment on the local, national and global levels [8]. This concept is not new but is experiencing an upsurge and has become increasingly popular within the past few years [9]. According to Robinson and colleagues, AMR is the quintessential “One Health” issue, as it is linked to all domains of life, especially with microbiology as its core [10]. *Campylobacter* spp. for example, is highly relevant in a “One Health” approach. Campylobacteriosis is the first cause of bacterial gastroenteritis in humans worldwide [11,12], where it occurs more frequently than infections caused by *Salmonella* spp., *Shigella* spp. and *Escherichia coli* O157:H57 [13,14]. Since the introduction of fluoroquinolones and macrolides as drugs of choice for the treatment of human gastroenteritis in the 1980s, many reports highlighted the emergence of resistance patterns within the *Campylobacter* genus. Likewise, recent studies reported the emergence of multi-resistant *Campylobacter* spp., to different classes of antibiotics from different sources [15,16,17,18]. Gölz and colleagues point out that a better understanding of the sources and pathways at the different stages of the food chain, thanks to a “One Health” approach, should allow better control and prevention of the *Campylobacter* burden in humans [19]. The overall understanding of the co-evolution dynamics between the three compartments is urgently needed to develop novel approaches to study AMR [9,10]. Mangioni and colleagues already highlighted the important need for the development of a “fast microbiology” era in diagnostics and especially in antimicrobial stewardship policies, resulting in a more rapid optimization of antimicrobial therapy, in order to improve patients handling and care [20]. The surveillance or quantification of AMR in all the different reservoirs is a challenging task as it requires complex tools [21]. In 2015, WHO launched a new surveillance program, called GLASS, for AMR monitoring of bacteria by regions, giving established guidelines to collect data for several restricted clinical pathogens and antibacterial classes [22]. Collecting data will be an important issue through antimicrobial susceptibility tests (AST) from diagnostic laboratories involved in the program. Hence, diagnostic laboratories are on the frontline for the detection of AMR, and they require fast and cost-effective tools for analysis. During the last decade, diagnostics underwent a real revolution with the advent of molecular biology techniques (e.g., DNA based-methods or proteomics), reducing the turn-around time [20]. However, the current “patient–physician” workflow (Figure 1) is still relatively long depending on the type of primary sample (e.g., blood, urine, stool or cerebrospinal fluid) and of the requirement for the full characterization of the pathogen, i.e., species/subspecies and AMR identification. Mass spectrometry may be considered as one of the main actors in the development of future fast microbiology technologies, as the method is already implemented in a majority of health care infrastructures for routine identification of microorganisms. 

The aim of this mini-review is to show how matrix-assisted laser desorption/ionization time of flight mass spectrometry (MALDI-TOF MS) could be handful for a fast combined species and AMR identification in enteric pathogens, by detecting specific biomarkers within protein spectra generated by MALDI-TOF MS. Likewise, the use of tandem mass spectrometry and bioinformatics as support tools for advanced identification of AMR will be discussed.

## 2. MALDI-TOF MS: A New Era for the Diagnostic Field

Current reference methods in routine laboratories for detection and identification of AMR, consist of antibiogram disk diffusion or microdilution tests and automated antibiograms (e.g., VITEK^®^ 2 apparatus from Biomérieux^©^). These approaches are time-consuming and require an incubation time between 12–24 h before the physician is able to prescribe the right cohort of antibiotics to the patient. In clinical research, molecular methods such as next-generation sequencing (e.g., whole-genome sequencing (WGS)) or nucleic acid based methods (e.g., polymerase chain reaction (PCR) techniques) are also used to detect and identify AMR genes [23]. However, even if PCR methods are already implemented in many clinical diagnostic and reference laboratories and there is a notable decrease of per-sample cost for WGS, their application in routine AMR surveillance especially in resource-limited countries is restricted [24].

In the field of biology, soft ionization mass spectrometry, such as MALDI-TOF MS, has been established for decades for the analysis of important biological molecules, such as proteins, peptides, oligonucleotides, lipids or glycans [25]. In 1975, Anhalt and Fenselau proved that mass spectrometry, coupled with pyrolysis, produced characteristic mass spectra for gram-negative bacteria [26]. The MALDI method was first introduced in biology in 1987 by Karas and colleagues, and followed by Tanaka and colleagues who were awarded a Nobel prize in chemistry “for their development of soft desorption ionization methods, for mass spectrometric analysis of biological macromolecules” [27,28,29]. With these findings and outcomes, growing interest in mass spectrometry and its application as a screening and diagnostic research tool has emerged [30]. In the last decade, MALDI-TOF MS has become popular in routine diagnostic laboratories and is now considered the new gold standard for the direct identification of microorganisms, and somehow revolutionized the microbiology field by progressively replacing all the biochemical (e.g., API gallery) and phenotypic tests [31] for species characterization. Despite the price of the MALDI-TOF MS apparatus, analyzing a full 96 MALDI target is virtually costless and only requires around 0.50 € of chemicals and consumables [32], and only requires a maximum time of 25 min to give 96 reliable species identifications. Commercial databases included with the device cover a large panel of bacteria [33], mycobacteria [29,34] and also fungi [35] of medical interest. In addition, several reports highlighted its successful application in other microbiology areas, for the identification of viruses [36], ectoparasites [37], protozoa [38] and helminths [39,40]. In clinical application, organisms isolated from different matrices (e.g., blood, urine, stool and cerebrospinal fluid), are applied directly on the target and covered by an acid reagent. Then the target is subjected to mass spectrometry for analysis, where a laser will shoot and ionize proteins that are separated by their mass-to-charge ratio (*m*/*z*) and analyzed by a detector. The signal will be translated into spectra, which will be compared with commercial or in-house databases and provide a rapid and reliable identification at a low cost and high precision (e.g., relevance score) [41].

**Figure 2:**
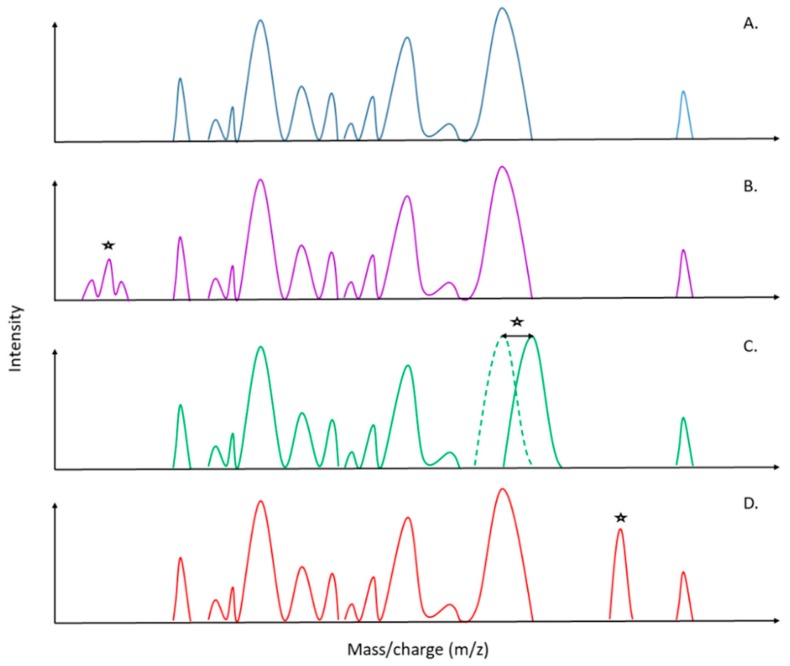
Schematic representation of possible MALDI-TOF MS spectra patterns for direct determination and identification of antimicrobial resistance. (**A**) Sensitive strain. (**B**) Detection of antimicrobial resistance by the detection of metabolites related to the degradation of the antibiotic. (**C**) Detection of antimicrobial resistance by the detection of a peak shift, which could be related to a mutation in the biomarker gene that confers antimicrobial resistance (AMR). (**D**) Detection of antimicrobial resistance by the detection of unique biomarkers, which could be related to the production of a specific molecule (e.g., enzymes, porins). (*) Peak differences in comparison with the sensitive strain spectra (**A**).

Since the introduction of mass spectrometry in the field of microbiology, the speed of pathogen identification has tremendously increased, thereby improving antimicrobial therapy, infection prevention and leading to a major impact in public health and epidemiology [42]. Today, direct antimicrobial resistance detection in the acquired mass spectra is one of the most suggested and asked about applications in specialized reviews [43,44,45,46,47]. Four main uses have been successfully tested: (1) the detection and expression of antibiotic resistance mechanisms (e.g., β-Lactamase, rRNA methyl-transferase activity), (2) specific mass peak profiles within spectra, (3) the detection of stable isotope-labeled biomarkers and (4) the estimation of the effect of antibiotics on microorganism growth. On one hand, the detection of antibiotic resistance mechanisms is the most explored method so far, as the degradation of antibiotics produces intracellular metabolites that generate specific peaks on spectra [48,49]. These peaks are directly visible on the spectra during analysis of the latter (Figure 2A,B). Nevertheless, those investigations still imply supplementary incubation time, yet less than for antibiograms, but are inherently further postponing the diagnosis to setting up an optimal antibiotherapy. Hence, the “patient–physician” workflow requires a concrete optimization for AMR detection with novel MALDI-TOF MS approaches, which is a special scope of this review. Identification of specific biomarkers within the protein spectra presents obvious advantages compared to other techniques (Figure 2C,D). Indeed, thanks to a unique spectrum, it will be possible to couple an accurate identification at the species/subspecies level as well as antimicrobial resistances only after a 25 min run of the MALDI-TOF (Figure 1 and Figure 2). It will drastically decrease workflow time, cost for diagnosis and hence, allow the physician to apply the effective cohort of antibiotics in an optimized time to the patient.

## 3. Specific Biomarkers as a Future Key for the Detection of AMR

In the clinical field, biomarkers are defined as biomolecules that are determined in a tissue or body fluid of a patient to identify a disease at the molecular level [50]. Developments of protein biomarker descriptions have been done for biological fluids, cell lines and solid tissues for many purposes like diagnosis, treatment, follow-up, etc. [50]. In mass spectrometry, a biomarker could be defined and identified as a specific unique peak, numerous peaks or a shift in the mass-to-charge ratio. Since the application of MALDI-TOF MS for the identification of microorganisms, only several publications remarked on its potential usefulness in detecting and characterizing antimicrobial resistances through specific biomarker(s) (Table 1). In 2000, Edwards-Jones and colleagues carried out the first work on the subject by noticing specific biomarkers, allowing the distinction between methicillin-sensitive (MSSA) and methicillin-resistant *Staphylococcus aureus* (MRSA) by intact cell mass spectrometry (ICMS), and concluded that ICMS could have the capacity to identify and perform typing of MRSA [51]. Their results were validated two years later by another group working on *S. aureus* [52], by also demonstrating a variation between the spectral profiles in the mass range of *m*/*z* 500–3500 Da.

Hindre and colleagues showed that it was possible to detect bacteriocins without specific purification from bacterial colonies, as lacticin, nisin and coagulin producing bacteria generate specific mass to charge ratio peaks for each molecule [53]. Additionally, Camara and Hays [54] differentiated wild-type *E. coli* from ampicillin-resistant plasmid-transformed *E. coli* strains by direct visualization of β-lactamase in the spectra. In 2011, another team reported the successful application of MALDI-TOF MS to differentiate between cfiA-positive and cfiA-negative *Bacteroides fragilis*, and hence their capacity to be potentially resistant to carbapenems, by the observation of a protein profile shift between the two different classes [55]. Currently the major avenue with MALDI-TOF MS is seeking specific peaks linked to porins [56], enzymes (e.g., VanA/B, mecA, KPC-2) [57,58,59,61,63] or even lipid modifications [62]. Furthermore, number of listed studies settle not only on the detection of specific biomarkers, but focus on processing parameters and creation of in-house databases, and therefore bioinformatics.

## 4. Bioinformatics: A Powerful Tool to Reinforce Diagnostics

Early automatic typing methods were mainly of a phenotypic nature (e.g., serotype or biochemical characteristics). However, with the advent of molecular biology, bioinformatics became unmissable and hence, a must in research to proceed and analyze genomic data in research. Bioinformatics can be defined as an interdisciplinary field developing methods and software tools for a better understanding of biological systems.

In diagnostics, dilution- or diffusion-based antibiograms are still currently the reference methods for phenotypic detection of AMR. With the emergence of new sequencing technologies, such as whole-genome sequencing (WGS), genomic data are more and more used for the identification and prediction of AMR thanks to the detection of specific sequences. Nowadays, different online user-friendly platforms able to use whole-genome data to extract relevant information, such as AMR genes, exist. The real advantage of these tools is that they are intended for scientists who do not necessarily have advanced bioinformatic skills. Many pipelines that are able to predict AMR patterns, such as Resfinder [65], AMRFinder [66], ARGS-OAP [67], SEAR [68] or ARGminer [69] are today online. Historically, Resfinder, developed by the Center for Genomic Epidemiology, was one of the first types of platforms of this kind, and it is a widely used AMR determinant detection program [65]. It is a web server that uses data for identifying acquired AMR genes and/or chromosomal mutations in total or partial sequenced isolates of bacteria, referring to nucleotide sequences from the National Center for Biotechnology Information (NCBI) databases (http://www.ncbi.nlm.nih.gov/nuccore/). Recently, NCBI developed a new tool, AMRFinder, using either protein annotations or nucleotide sequences to identify AMR genes. A first report comparing AMRFinder and Resfinder performance, using bacterial isolates from a collection from the U.S. AMR surveillance system program (NARMS) [66], highlights that incomplete or incorrect databases can lead to AMR misidentification. As an example, in some cases, where Resfinder generates a high scoring for an identification, the latter was incorrect due to the absence of a specific sequence in the database. However, the database issue is currently the same with MALDI-TOF MS for the identification of different species, with the results depending on the quality of the used database. Hence, even if online AMR detection platforms are useful to give a first glimpse of which AMR could be present, there is still a need to improve and implement databases with new and reliable sequences. For now these bioinformatics tools should be combined with phenotypic methods.

Mass spectrometers manufacturers, such as Bruker Daltonics^©^ (https://www.bruker.com/) propose software platforms (e.g., FlexControl™, FlexAnalysis™, Maldi Biotyper Compass Explorer™ and Clinpro Tools™) allowing the acquisition, processing of spectra and the creation of customized databases, and together with other bioinformatics pipelines provide new performant tools to the MALDI-TOF MS community [70,71]. Applied Maths NV^©^ (http://www.applied-maths.com/bionumerics), notably, proposes BioNumerics™, a pipeline platform for advanced analysis of spectra. It offers a large panel of competitive analysis applications, including fingerprinting, typing, MALDI spectrum processing and the creation of in-house databases, by the utilization of different default or customized modules [72]. Among the publications listed in Table 1, reports highlighted that spectrum-processing parameters (e.g., baseline subtraction and curve smoothing) increased the detection of AMR from *Campylobacter jejuni* [60]. Indeed, by applying optimized processing parameters, beta-lactam resistances detection was increased by 34%. Spectrum processing parameters should not be neglected and indeed enhance screening performance. Several other MALDI-TOF MS studies used BioNumerics™ as their main tool for analysis [73,74,75]. However, even if previously mentioned software suggests a high capacity to customize and optimize spectra, it is also important to highlight the fact that it is also possible to carry it out during the acquisition step by modifying MALDI-TOF parameters. Variables such as acquisition range (e.g., 2–20 kDa or 300 Da for the detection of antibiotic hydrolysis products), laser intensity, spectrum evaluation (e.g., peaks limit intensity) or ion source modifications (e.g., increase the resolution for low- and high-weight molecules), might be modified and adjusted. The combination of appropriate acquisition parameters and processing/optimization steps is key for MALDI-TOF spectra analysis and exploitation.

Various other software gives the opportunity to create and perform in-house databases. Jeverica and colleagues have successfully screened routine clinical *B. fragilis* isolates and determined their division (e.g., I or II), hence their potentiality to be resistant to carbapenems, thanks to the created in-house database of Nagy and colleagues [55,64]. Therefore, the creation of in-house databases, ideally sharing close experimental conditions and spectrum processing parameters should be the main avenues to be explored in the future, for the full optimization of the application of MALDI-TOF MS to detect AMR. In complement to commercial libraries, in-house, online or external databases exist and allow the comparison of user spectra. For example, the Centers for Disease Control and Prevention (CDC) curates a platform: MicrobeNet (https://microbenet.cdc.gov/), which is a free online database launched in 2013 with the goal to help clinical laboratories to improve their diagnostics. Moreover, they developed a collaboration with Bruker^©^, allowing users to search the database directly from the generated MALDI-TOF mass spectra. It is yet possible to match unknown acquired spectra to find out if someone else already identified it. As an example, a recent study [76] showed the application of external databases, such as SARAMIS™ (Spectral Archive and Microbial Identification System database) and PAPMID™ (Putative Assigned Protein Masses for Identification Database), and the 5800 TOF/TOF MALDI research instrument from Absciex^©^, as an efficient tool for the identification of 26 bacterial strains, with comparable accuracy to a commercial system. If the primary use of this online-database is widened to AMR thematics, it will be possible to share freshly discovered AMR biomarkers far more easily. In brief, bioinformatics offers a wide range of tools for the detection and identification of AMR, easily practicable in combination with MALDI-TOF MS.

## 5. MALDI-TOF/TOF Tandem Mass Spectrometry: To Infinity and Beyond

The development of soft-ionization methods such as MALDI or electrospray ionization (ESI) were important discoveries, as it was preserving the integrity of larger molecular weight compounds like proteins, carbohydrates or lipids [77]. MALDI-TOF MS would detect mainly ribosomal proteins, housekeeping proteins and structural proteins that are abundant in the cell, relatively independent of the growth state of the microorganism, in a mass range between 2 to 20 kDa [78]. However, this type of mass spectrometry is somehow self-limiting in its efficiency, depending on the mode used to give primary information, such as the mass of the analyzed compound [79]. Indeed, mass spectrometry technology presents different possible parameter adjustments, such as the linear (i.e., ion moves in a straight line from the source to detector) and reflectron (i.e., ion mirrors increasing the time of flight and the resolution) modes, or the investigation of positive and/or negative ions, to increase the resolution and selectivity of generated spectra [79]. The desire to know more than the mass of molecules brought up the development of complex mass spectrometers combining two analyzers (e.g., quadrupole, ion trap and TOF), called multi-analyzer systems or MS/MS [79]. The association of two identical types of analyzers is a tandem instrument. Among these tandem mass spectrometers, MALDI-TOF/TOF MS is commonly used in proteomic research, for the sequencing of peptides [80]. The first TOF analyzer serves as a mass filter [81], to select an ion of interest, whose corresponding fragment is communicated (or not) to the second analyzer [81]. High resolution and mass selectivity enable the identification of peptides, i.e., an individual biomarker from the protein, essential for the analysis of closely related species (or strains) or gene expression patterns [77]. However, fragmentation is only feasible for low mass weights (up to approximately 3 kDa), and if identified biomarkers have a higher mass, there will be a need to process through other MS approaches. As mentioned in previous sections, antimicrobial resistance can be targeted thanks to the presence of a specific peak related to the presence of enzymes, by peak shifting due to chromosomic mutation(s), and/or also by the presence of degradation molecules (Figure 2). The standard MALDI-TOF MS is able to detect such mechanisms. However, to know in precision which enzyme or mutation is involved in these specific mass-to-charge ratios, advanced analysis is required. In 2006, Pieper and colleagues carried out proteomic analyses of a sub-cellular fraction of *S. aureus* isolate VP32 with different resistances to the cell-wall targeting compound vancomycin [82,83]. They analyzed and determined significant protein abundance differences for 65 proteins by MALDI-TOF/TOF MS and liquid chromatography-MS/MS. Among these proteins, several enzymes involved in the biosynthesis of purines, peptidoglycan hydrolases and penicillin-binding proteins were identified. They concluded that different expression levels of these proteins might be responsible for structural changes of the peptidoglycan and hence conferring resistance to glycopeptide antibiotics. Such studies largely support the idea to link, in a close future, specific biomarkers detected by MALDI-TOF MS spectra to characteristic and often well-known biological phenotypic mechanisms.

However, until a MALDI-TOF MS spectrum could be able to give the utmost information at once, there is still a long way to go and issues can already be identified. First of all, before carrying out MALDI-TOF/TOF MS analysis, there is the need to identify a specific antimicrobial resistance biomarker. Nevertheless, if the biomolecules of interest, here an enzyme, is expressed in a low quantity by the cell, there are three possible limiting scenarios. The first one will be that MALDI-TOF MS does not detect it, due to too low intensity and hence no appearance on the spectra. The second one, the peak exists but the intensity is that low that during spectrum processing it could disappear. The last one, the specific peak will go through all the steps but would still have a too low intensity to be explored. An important point to mention is the resolution of the device itself. Indeed, manufacturers do not propose all the same resolution for their mass spectrometers. Most of the software used for the identification of spectra are working with three different components: (1) mode forward: How many peaks of the spectrum to be identified are present in the reference spectrum, (2) reverse mode: How many peaks in the spectrum of reference are present in the sample and (3) symmetry: Count the common peaks, and sum the intensity ratios. In this configuration, intensity is an important factor, whereas the frequency of apparition of peaks is not taken into account. As a suggestion, identification software should consider integrating into their algorithm a special mode dedicated to the calculus of peak frequency between the different analyzed spectra. Finally, there remains the question of the transition between the MALDI-TOF/TOF and MALDI-TOF spectra: Will it be possible to integrate specific biomarkers data from the MALDI-TOF/TOF spectra into a MALDI-TOF database? Indeed, the main objective for routine diagnostic laboratories will be to couple species identification, subtyping and antimicrobial resistance identification after the generation of one single spectrum. However, the detection of shifts due to the mutation of one or two bases in the genome requires high sensitivity and resolution. The integration of tandem TOF/TOF MS data will be ideal for the detection of such shifts, as the tandem technology has a higher setting than single MALDI-TOF MS. Straightaway, there is no report of a successful transfer of MALDI-TOF/TOF data through a MALDI-TOF system so far, which means there is still a specific need for further scientific and technological development. In the same line of thought, the cost of such a device and the development of specific skills for spectra analysis are currently still a serious stumbling block for its concrete implementation in diagnostics.

## 6. Outlook and Future Challenges for MALDI-TOF MS and AMR in the Diagnostic Field

During the last decade, antimicrobial resistance obviously became a serious issue for public health. However, international projects (e.g., EU-JAMRAI, EFFORT, JPIAMR, etc.) and challenging competitions (e.g., Antimicrobial Resistance Rapid, Point-of-Need Diagnostic Test-Challenge) have surfaced to find a way to reduce and/or optimize the use of antibiotics. Amongst others, the Longitude prize launched in 2014, with the aim to reward teams that can develop a cheap, accurate, rapid and easy-to-use point-of-care diagnostic test for bacterial infection, with a focus on antimicrobial resistance. In the context of developing a fast technology for diagnostics, much effort has been directed toward finding new alternatives for the detection of antimicrobial resistances implying MALDI-TOF MS as a new potential reference tool, and has now largely gone beyond the proof-of-concept stages [84]. The diagnostics mass spectrometry stage is mainly represented by the two manufacturers Bruker^©^ and Biomérieux^©^, which have largely contributed to the most recent innovation in terms of AMR detection by mass spectrometry. In one hand, Biomérieux^©^ proposes a complete automated identification (ID) /AST system, i.e., the VITEK^®^ SOLUTION (https://www.biomerieux-diagnostics.com/vitek-solutions), by coupling two of their devices: the MALDI-TOF VITEK^®^ MS, which furnishes the ID, and the VITEK^®^ 2 for AST. The ID/AST complex is supposed to give a result to clinicians within 14–20 h. On the other hand, during the ASM Microbe conference 2019 (www.asm.org), Bruker^©^ announced the launch of the MALDI Biotyper^®^ Sirius system [85], a versatile MALDI-TOF system for research purposes. It supports a novel negative/positive-ion switch mode assay for research and clinical studies in fast antibiotic resistance testing, such as colistin resistance in gram-negative bacteria [85]. Simultaneously, they introduced the MBT-STAR assay kit for detection of carbapenem and cephalosporin resistance. It measures the level of hydrolysis of the β-lactam ring after a 30 min incubation, thus providing a result within 60 min, after analysis by the MBT STAR-BL software module [85]. Finally Bruker^©^ developed a software module for subtyping antimicrobial resistances such as KPC-producing *K. pneumonia*, MRSA, and *B. fragilis* cfiA [86], inspired by the previously described studies in Table 1. According to the manufacturer, after a simple direct transfer on the target from the agar plate, the software will be able, after a high confidence identification, to process an automated typing (e.g., “presumptive KPC”, “presumptive PSM positive MRSA”) thanks to the detection of specific biomarkers [87]. However, much work still needs to be accomplished before exporting this technology to diagnostic and reference laboratories [84]. The detection of specific biomarkers in foodborne pathogens should give an advantage to obtaining the three-fold information within a single spectrum: species identification, sub-typing and antimicrobial susceptibility, to efficiently treat foodborne infections. The elaboration of in-house databases and processing parameters should be considered a key step to make MALDI-TOF MS a potential new gold standard for AMR detection.

The successful detection of specific antimicrobial resistance biomarkers on MALDI spectra within the same bacterial genus has been described in previous sections. However, a question still remains: could a specific AMR biomarker from one bacterial genus be applied and steadily transferred to another one? A working group detected the presence of biomarkers for the protein pKpQIL_p019, conferring carbapenem resistances in the *Enterobacteriaceae* family, in three different bacteria: *K. pneumoniae*, *E. coli* and *E. gergoviae*, at a mass-to-charge ratio of 11,109 m/z [87,88]. They specified the implementation of screening and analysis in the routine clinical workflow of their laboratory, with all spectra scanned by the automated script for peaks within a window of 11,109 ± 15 Da using Bruker^©^-provided platform software. By the creation of specific peak scripts peculiar for specific antimicrobial resistance, it is possible to detect antimicrobial mechanisms or resistances for different bacteria and to integrate these in a diagnosis workflow. However, this technique still needs to be explored for more antibiotics classes such as β-lactams, glycopeptides or macrolides.

Nowadays, WGS is considered as the current approach with the highest levels of discrimination in terms of subtyping, and studies have already reported its application as being effective to predict antimicrobial resistance in bacteria [89,90,91], and making it a valuable tool for antimicrobial resistance surveillance [23]. However, even if the sequencing price has significantly decreased during the past decade, this technology is not implemented in every diagnosis laboratory, and the analysis requires much more time than mass spectrometry. Yet still, very few studies show the tandem utilization of WGS and MALDI-TOF MS [92,93]. Both techniques present advantages and disadvantages but seem to show a particular complementarity. As an example, colony identification of *Elizabethkingia* spp., a ubiquitous bacteria found both in the environment and hospital settings, was carried out by MALDI-TOF MS [93]. WGS was used for the detection of antimicrobial resistance genes and to confirm MALDI-TOF MS identification. WGS showed a better identification rate than MALDI-TOF MS, due to the lack of reference spectra for *Elizabethkingia* spp. in MALDI-TOF MS commercial databases at the time of the study. They concluded that MALDI-TOF MS databases should be continuously updated and upgraded, while WGS proved to be a valuable tool for species identification confirmation and quite detailed characterization of multidrug-resistance. Further, a report [92] studied the usefulness of MALDI-TOF MS in an outbreak of vancomycin-resistant *Enterococcus faecium* in a hospital in comparison to WGS. They reported, due to multiple cluster types involved in the outbreak, that the cohort showed discrepancies between the two techniques. The authors highlighted MALDI-TOF MS limitations in this situation and suggested to study results carefully, while WGS can be used for determination of evolutionary distance between isolates. However, another important point to highlight, which is not mentioned in the latter studies, is that WGS is certainly able to accurately spot resistance genes, but it does not give any information on gene expression, while phenotype-based MALDI-TOF MS generates a spectrum based on protein expression and hence, gene expression. As MALDI-TOF MS is mainly used as a frontline tool in diagnostic laboratories, first results, such as species identification or AMR in the future, could be obtained rapidly, while species confirmation and antimicrobial resistance detection on the genome side could be obtained in a more delayed second step by WGS. MALDI-TOF MS and WGS should be seriously considered as complementary tandem tools and more studies should be led on this dual application.

*Escherichia coli*, *Staphylococcus aureus* and *Bacteroides fragilis* are the most MALDI-TOF MS studied enterobacteria according to Table 1. However, other enteric pathogens with a high impact incidence on human and animal health exist, which were not included in research reports so far. Lately, Batz and colleagues in their “Ranking the risk report” [94], list the three first bacterial foodborne pathogens as *Campylobacter* spp., *Salmonella* spp. and *Listeria monocytogenes*. Zautner and colleagues already reported the ability of MALDI-TOF MS to subtype *Campylobacter* spp. by shifts in biomarker masses, due to amino acid substitutions caused by single-point mutations in the respective biomarker gene [95], and they further described proteotyping as a promising tool for microbial typing at the species, subspecies, and even below subspecies levels [96,97,98]. These last studies show how generated spectra are exploitable and accurate enough to detect various AMR biomarkers in important pathogens such as *Salmonella* spp. or *Listeria* spp. Along the same line, carbapenems and β-lactams antibiotics families were the most tested and studied. However, gastroenteritis is the main end-up of a foodborne pathogen, and quinolones (e.g., ciprofloxacin) and macrolides (e.g., azithromycin and erythromycin) are the first frontline antibiotics used to treat such diseases [99]. Moreover, WHO categorized these two antibiotics as critically important [100] due to a high resistance prevalence concerning pathogens such as *Campylobacter* spp., *E. coli*, or non-typhoidal *Salmonella* spp. Nevertheless, at the moment there are no reports highlighting potential biomarkers for AMR to quinolones and macrolides. In a context where emerging multiple antimicrobial resistances are a critical issue, there is a need to collect data at least on these two antibiotic classes in order to ensure the collection, within one spectrum, of all the needed information.

Regarding our review on the detection of AMR by specific MALDI-TOF spectra patterns, there is still a lot to accomplish before MALDI-TOF MS could be considered the new reference method for the detection of antimicrobial resistance in routine diagnostics. Many questions still remain open and more studies should specifically be led on foodborne pathogens. Exploration on critical important antibiotics such as quinolones or macrolides, which are widely used for the treatment of foodborne illnesses, but unfortunately with no available data on it, should be of major interest for the scientific community. Finally, the dual combination of WGS and MALDI-TOF MS should soon become the main approach for the utmost reliable and fast identification of AMR in foodborne pathogens.

## Figures and Tables

**Table 1:** Specific whole-cell MALDI-TOF MS spectra patterns literature for identification of antimicrobial resistance in enteric bacteria.

Organism	Antibiotic Classes Tested	Biomarkers	Year	Reference
*Staphylococcus aureus*	β-lactams	MRSA: 891, 1140, 1165, 1229 and 2127 *m*/*z* MSSA: 2548 and 2647 *m*/*z*	2000	[51]
*Staphylococcus aureus*	β-lactams	Variation between in the spectral profiles in the mass range of *m*/*z* 500–3500 Da	2002	[52]
*Lactococcus lactis* *Bacillus coagulans* *Escherichia coli*	Bacteriocins (lantibiotic)	Lacticin 481: 2902, 2924,2940 *m*/*z* Nisin A: 3392 *m*/*z* Coagulin: 4650 *m*/*z*	2003	[53]
*Escherichia coli*	β-lactams	Ampicillin: 29.000 *m*/*z*	2007	[54]
*Bacteroides fragilis*	Carbapenems	cfiA negative: 4711, 4817, 5017, 5204, 5268 *m*/*z* cfiA positive: 4688, 4826, 5002, 5189, 5282 *m*/*z*	2011	[55]
*Klebsiella spp.*	Carbapenems	OmpK36 porin: 38000, 19000 *m*/*z*	2012	[56]
*Enterococcus faecium*	Glycopeptides	VanA/B: 6603 *m*/*z*	2012	[57,58]
*Enterobacteriaceae*	Carbapenems	bla_KPC_: 11109 *m*/*z*	2014	[59]
*Campylobacter jejuni*	β-lactams Tetracyclines Glycopeptides	Spectrum processing parameters increased the resistance detection	2016	[60]
*Staphylococcus aureus* *Staphylococcus epidermidis*	β-lactams	mecA: 2415 *m*/*z*	2016	[61]
*Escherichia coli*	Polymyxin	Lipid A modification: 1919 *m*/*z*	2018	[62]
*Klebsiella pneumonia Enterobacter cloacae Escherichia coli* *Serratia marcescens* *Citrobacter braakii, Pseudomonas aeruginosa*	Carbapenems	KPC-2: 28544 *m*/*z*	2019	[63]
*Bacteroides fragilis*	Carbapenems	Identification of *B. fragilis* with the validated “cfiA library” [55]	2019	[64]
